# Do Narcissists Enjoy Visiting Social Networking Sites? It Depends on How Adaptive They Are

**DOI:** 10.3389/fpsyg.2018.01739

**Published:** 2018-09-19

**Authors:** Yuanyuan Shi, Yu L. L. Luo, Ziyan Yang, Yunzhi Liu, Hanwushuang Bao

**Affiliations:** ^1^CAS Key Laboratory of Behavioral Science, Institute of Psychology, Chinese Academy of Sciences, Beijing, China; ^2^Department of Tourism, Fudan University, Shanghai, China; ^3^Department of Psychology, University of Chinese Academy of Sciences, Beijing, China

**Keywords:** narcissism, social networking sites (SNS), affective experience, emotion, self-esteem

## Abstract

Previous evidence suggests that narcissistic people tend to visit social networking sites (SNS) frequently, but the emotions accompanying their engagement on such sites has not been a significant subject of study. Therefore, we examined the relationship between narcissism and the affective experience on SNS in two different samples. To do so, we not only examined narcissism as a whole but also distinguished between adaptive and maladaptive narcissism. Results of the two studies consistently showed that: (1) narcissism as a whole was not correlated with the SNS affective experience; (2) maladaptive narcissism was predictive of a worse affective experience on SNS; and (3) partly due to a positive correlation with self-esteem, adaptive narcissism was associated with a better SNS affective experience. In addition, these findings held with SNS activities considered in simultaneity. The present research extends our understanding of the relationship between narcissism and social networking as well as that between emotion and social networking.

## Introduction

Nowadays, millions of people visit social networking sites (SNS) every day (CNNIC, 2017). Among active SNS users are narcissists (Gnambs and Appel, 2018). The term narcissism derives from Narcissus, a young man in Greek mythology who could only stare at his beautiful reflection in a pool. Narcissists^1^ in the modern age are far luckier than Narcissus. Owing to SNS, narcissists today can not only take and enhance their “selfie” images, but also display them to numerous people anywhere, anytime. Indeed, empirical research has found that narcissists overall are more active on SNS than non-narcissists (for reviews see, Liu and Baumeister, 2016; Gnambs and Appel, 2018).

Despite their high involvement with SNS, do narcissists truly enjoy using SNS? The answer to this question would enrich our understanding of narcissism in two ways. First, previous studies on narcissism and SNS have mostly focused on the behavioral part of SNS usage (e.g., Buffardi and Campbell, 2008; Liu and Baumeister, 2016), leaving the relationship between narcissism and the affective part of SNS usage (i.e., affect experienced while visiting SNS) unexplored. Second, in offline settings, narcissism is predicative of a better sense of well-being (Rose, 2002; Sedikides et al., 2004). As online life features more and more in the human experience, it is necessary to know whether narcissism also predicts well-being in this setting. To fulfill these aims, we examined the relationship between the affective experience on SNS and narcissism. Specifically, we targeted both narcissism as a whole and its two distinct facets, adaptive and maladaptive narcissism.

Narcissism is characterized by intrapersonal grandiosity, self-importance, and perceived uniqueness, as well as by interpersonal entitlement, exploitativeness, and exhibitionism (Morf and Rhodewalt, 2001; Brown et al., 2009). According to the self-regulatory processing model, to affirm and enhance their superiority, narcissists employ intra- and interpersonal strategies, such as biased self-evaluations and attention-capture behaviors (Morf and Rhodewalt, 2001). Narcissists’ needs for positivity and admiration can be well satisfied via SNS. In one way, narcissists can selectively post positive self-descriptions and selfies to affirm their own positive images (Bleske-Rechek et al., 2008; Winter et al., 2014). Narcissists can further take advantage of SNS to pursue connections with as many friends as possible and to show off what they are engaged in to a wide range of acquaintances (Bergman et al., 2011). Indeed, compared to non-narcissists, narcissists use SNS more frequently, have more friends, pursue more leisure interests, and upload more self-promotional contents (Buffardi and Campbell, 2008; Ong et al., 2011; Wang et al., 2012; Winter et al., 2014). In short, by targeting SNS activity, numerous studies have identified a positive connection between narcissism and SNS usage (Liu and Baumeister, 2016; Gnambs and Appel, 2018). This finding, however, is not the whole story.

Beyond the activities that SNS support, recent research has demonstrated that SNS usage also appeals to emotions or, SNS affective experiences (Wise et al., 2010; Krasnova et al., 2013). When visiting SNS, users not only initiate numerous activities but also experience various emotions^2^. A line of emerging studies has zeroed in on such emotions experienced during SNS visits. For example, consuming other people’s postings about happy events (e.g., vacation photographs) elicits feelings of envy (Krasnova et al., 2013). In addition to self-reports, physiological data (i.e., facial electromyography) have also shown that people experience emotional fluctuations when visiting SNS (Wise et al., 2010). Moreover, these feelings are consequential, with better affective experiences on SNS predicting higher offline psychological well-being. Such emotional associations with well-being are above and beyond any SNS activity (Shi et al., 2018). Given the relevance of SNS affective experience to psychological well-being, researchers have started to investigate personality factors that contribute to better SNS affective experience. A recent study showed that users with high agreeableness, extraversion, conscientiousness, and low neuroticism were likely to experience more positive emotions and less negative ones when visiting SNS (Shi et al., 2014).

As narcissism predisposes people to engage in SNS activity, would narcissism also predict people’s affective experience on SNS? SNS provide perfect platforms to show off and win over the admiration of others. Hence, it seems that narcissistic SNS users would derive satisfaction and happiness from such engagement. Some preliminary evidence, however, prompted us to question this assumption. Narcissists’ fragile high self-views often dictate extreme emotional reactivity to daily experiences (Rhodewalt et al., 1998). Compared to non-narcissists, narcissists tend to grow happier and more satisfied with positive occurrences yet more anxious and angrier when facing negative ones (Rhodewalt and Morf, 1998). In the context of SNS, if narcissistic users attract the attention they crave, their affective experience is likely to be positive. But what if they fail? A study of people’s responsiveness to narcissistic Facebook users showed that individuals high in narcissism were less likely to receive comments and “likes” in response to their status updates than individuals low in narcissism (Choi et al., 2015). Exploitativeness and entitlement, two maladaptive components of narcissism, mainly drove this effect. This dynamic suggests that narcissists, especially those who exhibit highly exploitative and entitled behaviors, may not receive as much attention as they expect, and consequently, may undergo many unpleasant feelings when using SNS.

In addition to situational influences, narcissistic SNS users’ moods may also depend on the composition of their personality. Various studies have identified a distinction between adaptive and maladaptive facets of narcissism (Watson and Biderman, 1993; Barry et al., 2003, 2007; Hepper et al., 2014; Cai et al., 2015)^3^. This distinction has been validated by using the seven-factor model of the Narcissistic Personality Inventory (NPI; Raskin and Terry, 1988), the primary measure of narcissism in over three quarters of social/personality research on narcissism (Cain et al., 2008). Factors such as authority (i.e., viewing oneself as a leader) and self-sufficiency (i.e., confidence in one’s abilities) characterize adaptive narcissism, whereas entitlement (i.e., a desire to be viewed as more important than others), exploitativeness (i.e., a willingness to achieve status over others), and exhibitionism (i.e., a need to be the center of attention and receive praise from others) are representative of maladaptive narcissism (Watson and Biderman, 1993; Barry et al., 2003). Adaptive and maladaptive narcissism differ from each other in personality correlates, associations with inter- and intra-personal adaptions, problem behaviors, developmental trajectories, and genetic and environmental foundations (for a review, see Cai and Luo, 2018). In particular, adaptive narcissism has been found to be associated with indicators of higher emotional well-being, such as assertiveness, self-esteem, low depression and anxiety. In contrast, maladaptive narcissism has been tied to signs of poorer emotional well-being, such as low self-esteem, high neuroticism, anxiety, and depression (Emmons, 1984; Watson and Biderman, 1993; Brown et al., 2009; Ackerman et al., 2011). Therefore, it is likely that the different connections of adaptive and maladaptive narcissism with emotional well-being would extend to SNS. This hypothesis needs to be tested. To determine whether narcissists enjoy using SNS, we carried out two cross-sectional studies. Study 1 examined the correlation between SNS affective experience and narcissism, and further distinguished between adaptive and maladaptive narcissism. We operationalized SNS affective experience as the frequencies of experiencing various positive and negative affect when surfing SNS (Shi et al., 2014). Following previous practice (Shi et al., 2014), we indexed SNS affective experience by balanced affect, i.e., a combination of both positive and negative affect. Balanced affect has been shown to be a better indicator of emotional well-being than either the positive or negative affect (Bradburn, 1969; Van Schuur and Kruijtbosch, 1995; Kim and Mueller, 2001), especially for people from interdependent cultures in East Asia, which emphasize a balance between positivity and negativity (Kitayama et al., 2000; Uchida et al., 2004).

Study 2 extended Study 1 by testing whether narcissism predicted SNS affective experience beyond SNS activity with a relatively large sample. Narcissism is related to various SNS activities (Liu and Baumeister, 2016), and these activities in turn relate to emotional outcomes (Toma, 2013; Burke and Kraut, 2016), including the online affective experience (Shi et al., 2014). It is possible that the relationship between adaptive/maladaptive narcissism and SNS affective experience is merely a byproduct of the connection between SNS activity and affect, or at least confounded by that connection. Hence, we controlled SNS activity in Study 2. Besides requiring basic information about SNS activity (e.g., frequency of SNS visiting) for our assessment, we divided various SNS activities into two groups based on the dual-factor model of SNS use: (1) self-presentation activities (e.g., updating photos and status), motivated by the need for self-presentation; and (2) social-interaction activities (e.g., comments and chats), driven by the need to belong (Nadkarni and Hofmann, 2012).

In keeping with previous findings that adaptive narcissism is associated with affective well-being while maladaptive narcissism is correlated with poor psychological well-being (Emmons, 1984; Watson and Biderman, 1993; Brown et al., 2009; Ackerman et al., 2011), we hypothesized that adaptive narcissism would predict better SNS affective experiences, whereas maladaptive narcissism would predict worse SNS affective experiences (*Hypothesis 1*). Given that narcissism as a whole includes both adaptive and maladaptive components, the combination of the two may then cancel each other out and result in a null correlation with SNS affective experience.

**FIGURE 1:**
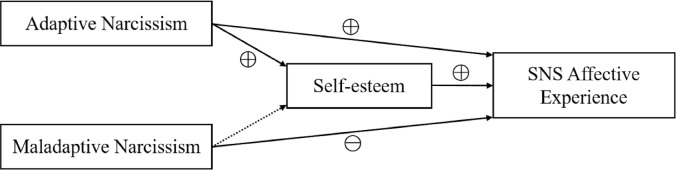
Conceptual model of how narcissism predicts SNS affective experience. Adaptive narcissism (solid line) is positively associated with self-esteem; self-esteem in turn increases SNS affective experience. Maladaptive narcissism also predicts SNS affective experience but in an inverse direction. We haven’t proposed a particular hypothesis for the relationship between maladaptive narcissism and self-esteem (dashed line).

In addition to identifying the correlation between adaptive narcissism and SNS affective experience, we hypothesized that self-esteem would play a role in this relationship (**Figure 1**). Self-esteem indicates the degree to which people feel good about themselves (Baumeister et al., 2003). Narcissists often possess high self-esteem (Campbell et al., 2002). But the self-views of narcissists and others with high self-esteem differ in many ways, including origins, development, content, and consequences (Campbell et al., 2002; Brummelman et al., 2016). Here, we included self-esteem as a potential mechanism underlying the link between adaptive narcissism and SNS affective experience for the following reasons: (1) narcissism, adaptive narcissism in particular, and self-esteem are closely related (Watson and Biderman, 1993; Bosson et al., 2008); (2) self-esteem is generally viewed as a positive indicator of well-being (DeNeve and Cooper, 1998; Baumeister et al., 2003); (3) self-esteem is associated with both SNS activity (Liu and Baumeister, 2016) and SNS affective experience (Shi et al., 2018); and (4) self-esteem accounts for the relationship between narcissism and well-being (Rose, 2002; Sedikides et al., 2004; Rosenthal and Hooley, 2010). Therefore, we expected self-esteem to account for the link between adaptive narcissism and SNS affective experience to some extent (*Hypothesis 2*). For maladaptive narcissism, we included it in the analysis but formulated no specific hypothesis. We based this decision on the small to negligible relationship observed to exist between self-esteem and maladaptive narcissism (Watson and Biderman, 1993; Ackerman et al., 2011).

## Study 1

### Methods

#### Participants

We recruited 205 university students from universities around the Institute of Psychology at the Chinese Academy of Sciences in Beijing. Four participants were excluded from the analysis as they reported not visiting SNS at all. The remaining 201 participants (98 males, 97 females, 6 unspecified; for the 198 participants who reported their age, *M_age_* = 21.59, *SD* = 2.90) reported that they accessed SNS regularly.

#### Procedure

The research protocol was approved by the Institutional Review Board (IRB) at the Institute of Psychology, Chinese Academy of Sciences. Informed written consent was obtained from all participants prior to survey. All participants completed a battery of assessments with paper and pencil in quiet classrooms. In the beginning, participants reported their frequency of using SNS (1 = *never*, 7 = *several times a day*) and completed measures of SNS affective experience, narcissism, and self-esteem. All measures were in Chinese. Translation and back-translation procedures were employed to ensure the equivalence across languages.

#### Measures

The *SNS Affective Experience Scale* consists of four negative (*unpleasant, sad, angry, afraid*) and four positive (*happy, pleasant, joyful, contented*) emotions, which are used to measure emotional well-being (Diener et al., 2010; Shi et al., 2014). Participants reported how often they experienced each affect while visiting SNS (1 = *never*, 7 = *always*). We averaged the scores across the eight emotions, with the scores of the negative items reversed. A higher mean score indicated an excess of positive over negative feelings.

The *Narcissistic Personality Inventory* (NPI, Raskin and Terry, 1988) comprises 40 items. Each item includes a pair of statements, one being narcissistic and the other non-narcissistic. For each item, participants indicated whether the narcissistic (coded as ‘1’) or non-narcissistic (coded as ‘0’) statement better described themselves. Reponses to all items were combined to serve as a measure of narcissism. Following previous practice (Barry et al., 2003, 2007; Hepper et al., 2014; Cai et al., 2015), 14 items (e.g., *I have a natural talent for influencing people*) reflecting self-sufficiency and authority were devised to index adaptive narcissism, while 18 items (e.g., *I would do almost anything on a dare*) addressing exhibitionism, exploitativeness, and entitlement were applied to assess maladaptive narcissism. We summed up the scores for every index, respectively.

The *Rosenberg Self-esteem Scale* (Rosenberg, 1965) includes 10 statements, like *“I take a positive attitude toward myself.*” Participants indicated the extent to which they agreed or disagreed with each statement on a 4-point Likert scale (1 = *strongly disagree*, 4 = *strongly agree*). We averaged the ten ratings to obtain an index of self-esteem.

### Results

**Table 1** shows the summary scores of and correlation coefficients among all measures. No correlation exists between the overall score of the NPI and SNS affective experience (**Table 1**). Because adaptive and maladaptive narcissism were correlated (**Table 1**), we conducted regression analyses to examine the unique predictive power of adaptive and maladaptive narcissism, with SNS affective experience as the outcome. The affective experience on SNS was positively related to adaptive narcissism, while negatively correlated with maladaptive narcissism (**Table 2**, Model 1). Notably, the significant relationship between adaptive narcissism and SNS affective experience was not evident in the zero-order correlation (**Table 1**) on account of suppression by maladaptive narcissism (Paulhus et al., 2004). As **Table 2** (Model 1) conveys, the direct effect of adaptive narcissism on SNS affect was positive. The indirect effect through maladaptive narcissism, however, should be negative, given the positive correlation between adaptive and maladaptive narcissism (**Table 1**) along with the negative effect of maladaptive narcissism on SNS affect (**Table 2**, Model 1). As predicted, the positive direct effect and the negative indirect effect canceled each other out, resulting in a null total effect (i.e., the zero-order correlation) of adaptive narcissism on SNS affect. Similarly, due to suppression by adaptive narcissism, the zero-order correlation between maladaptive narcissism and SNS affective experience was only marginally significant (**Table 1**).

**Table 1:** Summary scores of and correlations among all measures in Study 1.

Measure	*α*	*Mean^a^*	*SD*	1	2	3	4	5
(1) Narcissism	0.82	13.81	6.38					
(2) Adaptive narcissism	0.73	4.99	2.89	0.87^∗∗∗^				
(3) Maladaptive narcissism	0.65	5.86	3.07	0.87^∗∗∗^	0.60^∗∗∗^			
(4) Self-esteem	0.85	3.14	0.52	0.34^∗∗∗^	0.44^∗∗∗^	0.13^+^		
(5) SNS affective experience	0.63	5.10	0.72	-0.01	0.07	-0.13^+^	0.26^∗∗∗^	
(6) SNS use frequency	–	6.07	1.18	0.05	0.01	0.04	0.06	-0.02

^a^*For measures of narcissism, the means stand for the average of the summed items; ^+^*p* < 0.1; ^∗∗∗^*p* < 0.001*.

**Table 2:** Standardized coefficients (beta) of SNS affective experience regressed on narcissism, SNS use frequency, and self-esteem (Study 1).

Predictor	*β*	*p*
**Model 1**		
Adaptive narcissism	0.22	0.021
Maladaptive narcissism	-0.25	0.007
**Model 2**		
Adaptive narcissism	0.21	0.023
Maladaptive narcissism	-0.25	0.007
SNS use frequency	-0.05	0.484
**Model 3**		
Adaptive narcissism	0.07	0.483
Maladaptive narcissism	-0.21	0.026
SNS use frequency	-0.07	0.327
Self-esteem	0.25	0.002

As mentioned before, SNS activity could have confounded the relationship between adaptive and maladaptive narcissism and SNS affect. To account for this possibility, we considered the frequency of SNS use as an additional predictor of SNS affect (**Table 2**, Model 2). We found that the correlations between SNS affect and either adaptive narcissism or maladaptive narcissism did not change after all. In addition, there was no significant relation between SNS use frequency and SNS affect.

As self-esteem was correlated with both adaptive narcissism and SNS affective experience (**Table 1**), we entered self-esteem as another predictor in the above mentioned regression model (**Table 2**, Model 3) in order to test whether it would account for the relationship between adaptive narcissism and SNS affective experience. The results showed that when self-esteem was included in the regression model, the relationship between adaptive narcissism and affective experience became non-significant. Also, we conducted bootstrap analysis to calculate bias-corrected confidence intervals to evaluate the indirect effect (5,000 bootstrap resamples; Hayes, 2013). Our analysis yielded a significant indirect effect, with a point estimate of 0.03 and 95% bootstrap confidence interval that does not include zero [0.01, 0.05]. Therefore, self-esteem played a role in the connection between adaptive narcissism and SNS affective experience. In contrast, the relationship between maladaptive narcissism and SNS affective experience remained almost the same when self-esteem was introduced into the regression model, indicating that the effect of maladaptive narcissism was independent from self-esteem. Please see the **Supplementary Material** for results of additional analyses.

To summarize, our study supported *Hypothesis 1*. Narcissism as a whole was not related to affect experienced on SNS. But the adaptive facet of narcissism positively predicted SNS affective experience, whereas maladaptive narcissism negatively predicted SNS affective experience. In addition, self-esteem accounted for the correlation between adaptive narcissism and SNS affect, supporting *Hypothesis 2*. To replicate the results and further rule out potential influence from various SNS activities, we conducted another study with a larger sample.

## Study 2

In Study 2, we intended to replicate the results from Study 1. Furthermore, we controlled for the potential confounding effects of SNS activity, which has been related to both narcissism and affective experience on SNS (Shi et al., 2014; Liu and Baumeister, 2016).

### Methods

#### Participants

We recruited 464 young adults from the Beijing Twin Study (BeTwiSt). Twins in the BeTwiSt are socio-demographically representative of their peers in Beijing, including both college students and young employees (Chen et al., 2013). Fifty-six participants were excluded from the analysis as they stated they did not visit SNS at all. The remaining 408 participants (183 males, 225 females; *M_age_* = 20.34, *SD* = 1.86) reported that they accessed SNS regularly.

#### Procedure

The research protocol was approved by the Institutional Review Board (IRB) at the Institute of Psychology, Chinese Academy of Sciences. Informed written consent was obtained from all participants prior to survey. Participants completed all measures on personal computers in quiet, private rooms.

#### Measures

We assessed narcissism, self-esteem, and SNS affective experience with the same measures used in Study 1. We also examined SNS activities with the *SNS Feature Usage Scale* (Shi et al., 2014), which was developed based on earlier work (Ross et al., 2009; Ryan and Xenos, 2011; Nadkarni and Hofmann, 2012). The scale contains 12 items addressing different types of SNS activities, namely, general use, self-presentation, and social interaction (**Table 3**). Participants were instructed to indicate their frequency in participating in each activity based upon the usage of their favorite SNS. To overcome a major limitation of previous studies, reliance on results from a single-item measure for each specific use of SNS (Bergman et al., 2011), we composed criterion variables by averaging scores for each category.

**Table 3:** Social networking sites feature usage scale.

**General use**
How often do you visit SNS?
How long do you stay on SNS for each visit on average?^a^
How many friends do you have on your favorite SNS?^b^
**Self-presentation**
How often do you write notes/blogs?
How often do you update your profile image?
How often do you post photos?
How often do you update your status?
**Social interaction**
How often do you send private messages to others?
How often do you share or re-send others’ profiles?
How often do you visit your friends’ homepage?
How often do you comment on others’ posts?
How often do you check others’ comments on your posts?

*Response scale: 1 = never, 7 = several times a day. ^*a*^Response scale: 1 = less than 15 min, 7 = more than 4 h. ^*b*^Response scale: 1 = 1–50, 7 = over 500*.

#### Hierarchical Linear Modeling

Our data were dyadic and thus might have introduced bias in many parametric analyses by violating the common assumption of independence (Bliese and Hanges, 2004). To test for the presence of non-independence, we used the intra-class correlation coefficient (ICC), which suggested inter-dependence for twin members on all the measures (ICCs: 0.329 – 0.489, ps < 0.001). We further conducted hierarchical linear modeling (HLM) to analyze the dyadic data (Kenny et al., 2006). We ran these models based on the MIXED procedure in SPSS 23.0 (REML as estimation method, compound symmetry as covariance structure), with SNS affective experience as the outcome variable and adaptive and maladaptive narcissism as the level-1 predictors. This procedure estimates non-independence within dyads as a covariance. To control for SNS activity and self-esteem, we ran models with SNS activity and self-esteem as additional level-1 predictors. Finally, we estimated the indirect effect of adaptive narcissism on SNS affective experience via self-esteem, by using a Monte Carlo macro in SPSS (MCMED; Hayes, 2013).

### Results

As in Study 1, we found no significant relationship between overall narcissism and SNS affective experience (**Table 4**). As participants were nested within twin pairs, we used HLM to examine the unique predictive effects of adaptive and maladaptive narcissism on SNS affect. The affective experience on SNS was positively related to adaptive narcissism, while negatively correlated with maladaptive narcissism (**Table 5**, Model 1). Also similar to Study 1, suppression effects existed. The negative correlation between maladaptive narcissism and SNS affective experience was not evident in the zero-order correlation (**Table 4**). That is, the negative direct effect of maladaptive narcissism on SNS affect (**Table 5**, Model 1) and the positive indirect effect of maladaptive narcissism on SNS affect via adaptive narcissism canceled each other out.

**Table 4:** Summary scores of and correlations among all measures in Study 2.

Measure	*α*	*Mean^a^*	*SD*	1	2	3	4	5	6	7
(1) Narcissism	0.81	13.09	6.05							
(2) Adaptive narcissism	0.62	4.80	2.47	0.79^∗∗∗^						
(3) Maladaptive narcissism	0.67	5.79	3.09	0.87^∗∗∗^	0.49^∗∗∗^					
(4) Self-esteem	0.78	3.17	0.43	0.32^∗∗∗^	0.37^∗∗∗^	0.14^∗∗^				
(5) General use of SNS	0.60	3.45	1.39	0.12^∗^	0.00	0.18^∗∗∗^	0.04			
(6) Social interaction on SNS	0.81	4.04	1.37	0.03	-0.01	0.07	0.01	0.38^∗∗∗^		
(7) Self-presentation on SNS	0.66	2.77	0.97	0.12^∗^	0.04	0.19^∗∗^	-0.01	0.33^∗∗∗^	0.54^∗∗∗^	
(8) SNS affective experience	0.80	5.08	0.77	0.09^+^	0.17^∗∗^	-0.04	0.33^∗∗∗^	0.06	0.10^+^	0.01

^a^*For measures of narcissism, the means stand for the average of the summed items; ^+^*p* < 0.1; ^∗^*p* < 0.05; ^∗∗^*p* < 0.01; ^∗∗∗^*p* < 0.001*.

**Table 5:** Standardized coefficients (beta) of SNS affective experience regressed on narcissism, self-esteem, and SNS feature usage (Study 2).

Predictor	*β*	*p*
**Model 1**		
Adaptive narcissism	0.23	0.000
Maladaptive narcissism	-0.13	0.018
**Model 2**		
Adaptive narcissism	0.24	0.000
Maladaptive narcissism	-0.14	0.011
General use in SNS	0.06	0.267
Social interaction in SNS	0.10	0.070
Self-presentation in SNS	-0.05	0.375
**Model 3**		
Adaptive narcissism	0.13	0.017
Maladaptive narcissism	-0.13	0.014
General use in SNS	0.05	0.361
Social interaction in SNS	0.10	0.063
Self-presentation in SNS	-0.04	0.464
Self-esteem	0.28	0.000

*HLM equations for the three models: Model 1: Level-1 equation, Y_*ij*_ = *β*_*0*_ + *β*_*1*_^∗^adaptive narcissism + *β*_*2*_^∗^maladaptive narcissism + r_*ij*_; Level-2 equation, *β*_*0*_ = γ_*00*_ + u_*0j*_. Model 2: Level-1 equation, Y_*ij*_ = *β*_*0*_ + *β*_*1*_^∗^adaptive narcissism + *β*_*2*_^∗^maladaptive narcissism + *β*_*3*_^∗^general use + *β*_*4*_^∗^social interaction + *β*_*5*_^∗^self-presentation + r_*ij*_; Level-2 equation, *β*_*0*_ = γ_*00*_ + u_*0j*_. Model 3: Level-1 equation, Y_*ij*_ = *β*_*0*_ + *β*_*1*_^∗^adaptive narcissism + *β*_*2*_^∗^maladaptive narcissism + *β*_*3*_^∗^general use + *β*_*4*_^∗^social interaction + *β*_*5*_^∗^self-presentation + *β*_*6*_^∗^self-esteem + r_*ij*_; Level-2 equation, *β*_*0*_ = γ_*00*_ + u_*0j*_. We use the equations of Model 1 as an example to explain the meaning of these equations. An individual’s score (Y_*ij*_) is a function of (1) the grand mean, γ_*00*_; (2) group j’s deviation from the grand mean (level-2 effect), u_*0j*_; (3) fixed effects by adaptive and maladaptive narcissism, *β*_*1*_ and *β*_*2*_ (level-1 effect); (4) plus an individual i’s deviation from the group mean, r_*ij*_ (level-1 effect)*.

Extending the reach of Study 1, we tested whether adaptive and maladaptive narcissism could still predict SNS affective experience when considering various SNS activities in simultaneity. Indeed, when we included the measures of SNS activity in the HLM (**Table 5**, Model 2), the correlations between the two facets of narcissism and the SNS affective experience remained the same. Moreover, none of the correlations between any SNS activities and SNS affective experience was significant (*p*s > 0.05).

Again like Study 1, self-esteem was related to both adaptive narcissism and SNS affective experience (**Table 4**). To test the indirect effect due to self-esteem, we entered self-esteem as another predictor in the HLM. The correlation between adaptive narcissism and SNS affective experience decreased, albeit still significant (**Table 5**, Model 3). As a final step, we tested the indirect effect of adaptive narcissism on SNS affective experience via self-esteem and calculated a 95% confidence interval (CI) by using a Monte Carlo SPSS macro (based on 10,000 Monte Carlo samples; Hayes, 2013). The analysis revealed a significant indirect effect as 0.03 (95% CI [0.02, 0.05]). Hence, the results indicated that self-esteem accounted for part of the correlation between adaptive narcissism and SNS affective experience. Notably, like Study 1, the relationship between maladaptive narcissism and the affective experience was not due to self-esteem as their correlation did not decrease when self-esteem was included in the HLM. Please see the **Supplementary Material** for results of additional analyses.

To sum up, Study 2 verified the hypothetical differential relationship between the two facets of narcissism and affect experienced on SNS, replicating the results of Study 1 and supporting *Hypotheses* 1 and 2. Moreover, this study showed that these hypotheses held their ground when various SNS activities were simultaneously taken into account.

## General Discussion

To date, a large number of studies have demonstrated that narcissistic people use SNS more frequently and extensively than non-narcissistic people do. But few studies have examined whether narcissists genuinely enjoy using SNS. The present research suggested that the answer depends on the degree of adaptiveness of a particular individual’s narcissism. That is, narcissism as a whole does not predict the SNS affective experience. Instead, its two facets, adaptive and maladaptive narcissism, predict better and worse SNS affective experiences, respectively.

### Implications

The above findings have several implications. Emerging research has shown that the affect accompanying SNS use is an important part of a user’s online experience, which in turn may relate to the individual’s psychological well-being offline (Wise et al., 2010; Krasnova et al., 2013; Shi et al., 2018). Such affect experienced on SNS may be predicated by an individual’s personality, i.e., the Big Five factors (Shi et al., 2014). Beyond general personality traits, our results, for the first time, revealed that narcissism could also predict an individual’s feelings while visiting SNS. Specifically, adaptive narcissism was associated with more positive affective experiences, whereas maladaptive narcissism was correlated with more negative affective experiences. In other words, adaptive narcissists would more likely enjoy using SNS, whereas maladaptive narcissists may not. This finding is consistent with and enhances previous observations of differential relationships between the two facets of narcissism and emotional well-being (Watson and Biderman, 1993; Brown et al., 2009; Ackerman et al., 2011). Combined with existing findings, it is evident that adaptive and maladaptive narcissism are relevant to emotional well-being both online and offline. Given the potential link between SNS affective experience and psychological well-being offline (Krasnova et al., 2013; Shi et al., 2018), our results also suggested that any SNS benefits to the psychological well-being of narcissistic users may also depend on the adaptiveness of their personality traits. Ongoing research is necessary to confirm this supposition.

To understand the nuanced relationship between different facets of narcissism and the SNS affective experience, we explored the role of self-esteem in the link between SNS affective experience and narcissism, especially for adaptive narcissism. Indeed, we found that self-esteem partly accounted for the positive relationship between adaptive narcissism and the SNS affective experience. This finding corresponds with previous reports that self-esteem can explain the connection between narcissism and emotional well-being (Rose, 2002; Sedikides et al., 2004). Our results imply that adaptively narcissistic SNS users could experience more positive affect when surfing SNS, especially when they have high self-esteem. Meanwhile, the effect of self-esteem on the SNS affective experience was no less, or even larger, than the effects of adaptive and maladaptive narcissism. This indicates that people of high self-esteem could enjoy visiting SNS regardless of their narcissistic levels, which corresponds to the role of self-esteem in predicting offline well-being (DeNeve and Cooper, 1998; Baumeister et al., 2003). Taken together, these implications echo “the-rich-get-richer” theory, which posits that socially skilled people gain more social benefits from SNS (Amichai-Hamburger et al., 2008). In our case, this notion could be articulated as *the-happy-get-happier*. That is, people endorsing a positive self-view, which indicates psychological well-being in offline settings (Taylor and Brown, 1988), could gain a greater emotional boost from SNS and hence become happier. This effect applies to both adaptive narcissists and high-self-esteem individuals, though their self-positivity differs in various ways (Campbell et al., 2002; Brummelman et al., 2016). Our findings expand on previous research that shows SNS activity could increase (or decrease) users’ well-being (Toma and Hancock, 2013; Burke and Kraut, 2016), and highlight that the potential for SNS to bring happiness depends not only on how they are used but also on who use them.

In examining the relationship between narcissism and SNS, the majority of past studies treated narcissism as a single construct and produced inconsistent findings (Buffardi and Campbell, 2008; Bergman et al., 2011; Wang et al., 2012; Große Deters et al., 2014). Our finding of a null correlation between overall narcissism and the SNS affective experience but meaningful connections between different facets of narcissism and the SNS affective experience suggests that the inconsistency in the literature may be due to the fact that narcissism is not a unified construct. In addition, the suppression effects observed in the analyses not only justify the necessity of differentiating between adaptive and maladaptive narcissism but also highlight the importance of looking beyond mere correlations to understand the relationship between narcissism subcomponents and the SNS affective experience. Moreover, as the present study concerns emotional adjustment, the adaptive-maladaptive differentiation makes better sense than other models and components. The multidimensional nature of narcissism, nevertheless, leaves open the possibility that we can explore the relationship between various facets of narcissism and SNS usage across diverse approaches, for example, distinguishing between grandiose and vulnerable narcissism (Casale et al., 2016), or covert and overt narcissism (Ljepava et al., 2013). To be sure, the complexity of narcissism and SNS usage warrants more studies.

### Limitations and Future Research

The present studies were limited in several ways. The first limitation pertains to the cross-sectional design in our studies. The correlational methodology cannot infer causation across narcissism, self-esteem, and SNS affective experience. To examine the possible causal relationship among them, future research should employ an experimental or longitudinal paradigm. Second, we assessed SNS affective experience on the basis of retrospective self-reports, which might involve memory errors or/and be influenced by a participant’s current mood. To rule out these potential confounding factors, future studies would benefit from more objective and sophisticated measures, for instance, objective SNS page coding (Buffardi and Campbell, 2008). Third, in assessing SNS activities, we assessed general engagement with SNS, which combines SNS activities involving different communication types (e.g., active vs. passive) and targeting different audiences (e.g., close friends vs. acquaintances; Kraut and Burke, 2015). Diverse SNS activities have differential impact on well-being (Toma and Hancock, 2013; Burke and Kraut, 2016). Mixing divergent activities may hinder detecting the main effects of SNS activities on affective outcomes and examining the interactions between SNS activities and narcissistic personality traits. Thus, differentiating SNS activities according to communication types and audience would help to reveal a more nuanced reading of narcissists’ emotions that result from SNS use. For example, people high in adaptive narcissism might feel good when engaging in active activities, whereas people high in maladaptive narcissism would not, and both groups might feel less happy during passive activities.

Another limitation concerns the measures of adaptive and maladaptive narcissism. Although adaptive and maladaptive narcissism are distinct from each other in many ways, the scores of their measures revealed a medium correlation in both studies. This partial overlap is rooted in current conceptualizations and operationalizations of adaptive and maladaptive narcissism, which are based almost exclusively on reformulations of the NPI (Cai and Luo, 2018). This issue, however, is beyond the scope of the present research. Future replications are needed when new measures for these two facets of narcissism are developed. Finally, the present studies only examined narcissism in the context of predicting SNS affective experience. Some other personality traits, such as psychopathic factors (e.g., depression, anxiety, and loneliness), are associated with both narcissism (e.g., Tritt et al., 2010; Clarke et al., 2015) and SNS use (e.g., Ceyhan and Ceyhan, 2008; Koc and Gulyagci, 2013; Kross et al., 2013). They might also serve as predictors of online affective experience.

## Conclusion

Social networking sites are becoming indispensable to billions of people. Hence, it is crucial to ascertain whether and how they can benefit people’s psychological well-being. The current research augments extant literature by demonstrating the role of adaptive and maladaptive narcissism in predicting emotions that individuals can experience while using SNS.

## Ethics Statement

This study was carried out in accordance with the recommendations of Institute of Psychology, Chinese Academy of Sciences with written informed consent from all subjects. The protocol was approved by Institute of Psychology, Chinese Academy of Sciences.

## Author Contributions

YS and YLLL designed the study. ZY, HB, and YL collected the data. All the authors contributed substantially to data analysis and manuscript writing, provided final approval of the version to be published, and agree to be accountable for all aspects of the work in ensuring that questions related to the accuracy or integrity of any part of the work are appropriately investigated and resolved.

## Conflict of Interest Statement

The authors declare that the research was conducted in the absence of any commercial or financial relationships that could be construed as a potential conflict of interest.

## Footnotes

1 For the convenience of terminology, we use the expression *narcissist* to refer to a person exhibiting relatively high subclinical narcissism.

2 These emotions might be the causes or outcomes of the activities (Kross et al., 2013; Toma, 2013; Toma and Hancock, 2013). Emotions and activities may also occur in simultaneity (Wise et al., 2010). While also important, the relationship between SNS emotions and activities is beyond the scope of this study.

3 These terms refer to the relative social desirability of the two facets. Both adaptive and maladaptive facets may serve intrapersonal functions for narcissists, and neither reflects vulnerability, pathology, or disorder (Hepper et al., 2014).

## References

[B1] Ackerman R. A. Witt E. A. Donnellan M. B. Trzesniewski K. H. Robins R. W. Kashy D. A. (2011). What does the narcissistic personality inventory really measure? *Assessment* 18 67–87. 10.1177/1073191110382845 20876550

[B2] Amichai-Hamburger Y. Kaplan H. Dorpatcheon N. (2008). Click to the past: the impact of extroversion by users of nostalgic websites on the use of Internet social services. *Comput. Hum. Behav.* 24 1907–1912. 10.1016/j.chb.2008.02.005

[B3] Barry C. T. Frick P. J. Adler K. K. Grafeman S. J. (2007). The predictive utility of narcissism among children and adolescents: evidence for a distinction between adaptive and maladaptive Narcissism. *J. Child Fam. Stud.* 16 508–521. 10.1007/s10826-006-9102-5

[B4] Barry C. T. Frick P. J. Killian A. L. (2003). The relation of narcissism and self-esteem to conduct problems in children: a preliminary investigation. *J. Clin. Child Adolesc. Psychol.* 32 139–152. 10.1207/S15374424JCCP3201_13 12573939

[B5] Baumeister R. F. Campbell J. D. Krueger J. I. Vohs K. D. (2003). Does high self-esteem cause better performance, interpersonal success, happiness, or healthier lifestyles? *Psychol. Sci. Public Int.* 4 1–44. 10.1111/1529-1006.01431 26151640

[B6] Bergman S. M. Fearrington M. E. Davenport S. W. Bergman J. Z. (2011). Millennials, narcissism, and social networking: what narcissists do on social networking sites and why. *Pers. Individ. Dif.* 50 706–711. 10.1016/j.paid.2010.12.022

[B7] Bleske-Rechek A. Remiker M. W. Baker J. P. (2008). Narcissistic men and women think they are so hot–But they are not. *Pers. Individ. Dif.* 45 420–424. 10.1016/j.paid.2008.05.018

[B8] Bliese P. D. Hanges P. J. (2004). Being both too liberal and too conservative: the perils of treating grouped data as though they were independent. *Organ. Res. Methods* 7 400–417. 10.1177/1094428104268542

[B9] Bosson J. K. Lakey C. E. Campbell W. K. Zeigler-Hill V. Jordan C. H. Kernis M. H. (2008). Untangling the links between narcissism and self-esteem: a theoretical and empirical review. *Soc. Pers. Psychol. Comp.* 2 1415–1439. 10.1111/j.1751-9004.2008.00089.x

[B10] Bradburn N. M. (1969). *The Structure of Psychological Well-being.* Chicago, IL: Aldine.

[B11] Brown R. P. Budzek K. Tamborski M. (2009). On the meaning and measure of narcissism. *Pers. Soc. Psychol. Bull.* 35 951–964. 10.1177/0146167209335461 19487486

[B12] Brummelman E. Thomaes S. Sedikides C. (2016). Separating narcissism from self-esteem. *Curr. Dir. Psychol. Sci.* 25 8–13. 10.1177/0963721415619737

[B13] Buffardi L. E. Campbell W. K. (2008). Narcissism and social networking web sites. *Pers. Soc. Psychol. Bull.* 34 1303–1314. 10.1177/0146167208320061 18599659

[B14] Burke M. Kraut R. E. (2016). The relationship between facebook use and well-being depends on communication type and tie strength. *J. Comput. Med. Commun.* 21 265–281. 10.1111/jcc4.12162

[B15] Cai H. Luo Y. L. L. (2018). “Distinguishing between adaptive and maladaptive narcissism,” in *The Handbook of Trait Narcissism: Key Advances, Research Methods, and Controversies*, eds Hermann T. Brunell A. B. Foster J. (Berlin: Springer).

[B16] Cai H. Shi Y. Fang X. Luo Y. L. L. (2015). Narcissism predicts impulsive buying: phenotypic and genetic evidence. *Front. Psychol.* 6:881 10.3389/fpsyg.2015.00881 PMC4493767 26217251

[B17] Cain N. M. Pincus A. L. Ansell E. B. (2008). Narcissism at the crossroads: phenotypic description of pathological narcissism across clinical theory, social/personality psychology, and psychiatric diagnosis. *Clin. Psychol. Rev.* 28 638–656. 10.1016/j.cpr.2007.09.006 18029072

[B18] Campbell W. K. Rudich E. A. Sedikides C. (2002). Narcissism, self-esteem, and the positivity of self-views: two portraits of self-love. *Pers. Soc. Psychol. Bull.* 28 358–368. 10.1177/0146167202286007

[B19] Casale S. Fioravanti G. Rugai L. (2016). Grandiose and vulnerable narcissists: who is at higher risk for social networking addiction? *Cyberpsychol. Behav. Soc. Netw.* 19 510–515. 10.1089/cyber.2016.0189 27362922

[B20] Ceyhan A. A. Ceyhan E. (2008). Loneliness, depression, and computer self-efficacy as predictors of problematic internet use. *Cyberpsychol. Behav.* 11 699–701. 10.1089/cpb.2007.0255 19072150

[B21] Chen J. Li X. Zhang J. Natsuaki M. N. Leve L. D. Harold G. T. (2013). The Beijing Twin Study (BeTwiSt): a longitudinal study of child and adolescent development. *Twin Res. Hum. Genet.* 16 91–97. 10.1017/thg.2012.115 23177327

[B22] Choi M. Panek E. T. Nardis Y. Toma C. L. (2015). When social media isn’t social: friends’ responsiveness to narcissists on Facebook. *Pers. Individ. Dif.* 77 209–214. 10.1016/j.paid.2014.12.056

[B23] Clarke I. E. Karlov L. Neale N. J. (2015). The many faces of narcissism: narcissism factors and their predictive utility. *Pers. Individ. Dif.* 81 90–95. 10.1016/j.paid.2014.11.021

[B24] CNNIC (2017). *Statistical Report on Internet Development in China.* Available at: http://www.cnnic.net.cn/hlwfzyj/hlwxzbg/hlwtjbg/201708/P020170807351923262153.pdf

[B25] DeNeve K. M. Cooper H. (1998). The happy personality: a meta-analysis of 137 personality traits and subjective well-being. *Psychol. Bull.* 124 197–229. 10.1037/0033-2909.124.2.197 9747186

[B26] Diener E. Wirtz D. Tov W. Kim-Prieto C. Choi D.-W. Oishi S. (2010). New well-being measures: short scales to assess flourishing and positive and negative feelings. *Soc. Indicat. Res.* 97 143–156. 10.1007/s11205-009-9493-y

[B27] Emmons R. A. (1984). Factor analysis and construct validity of the narcissistic personality inventory. *J. Pers. Assess.* 48 291–300. 10.1207/s15327752jpa4803_11 16367528

[B28] Gnambs T. Appel M. (2018). Narcissism and social networking behavior: a meta-analysis. *J. Pers.* 86 200–212. 10.1111/jopy.12305 28170106

[B29] Große Deters F. Mehl M. R. Eid M. (2014). Narcissistic power poster? On the relationship between narcissism and status updating activity on Facebook. *J. Res. Pers.* 53 165–174. 10.1016/j.jrp.2014.10.004

[B30] Hayes A. F. (2013). *Introduction to Mediation, Moderation, and Conditional Process Analysis: A Regression-Based Approach.* New York, NY: Guilford Press.

[B31] Hepper E. G. Hart C. M. Sedikides C. (2014). Moving narcissus: can narcissists be empathic? *Pers. Soc. Psychol. Bull.* 40 1079–1091. 10.1177/0146167214535812 24878930

[B32] Kenny D. A. Kashy D. A. Cook W. L. (2006). *Dyadic Data Analysis.* New York, NY: Guilford Press.

[B33] Kim K. A. Mueller D. J. (2001). To balance or not to balance: confirmatory factor analysis of the affect-balance scale. *J. Happiness Stud.* 2 289–306. 10.1023/A:1013519931082

[B34] Kitayama S. Markus H. R. Kurokawa M. (2000). Culture emotion and well-being: good feelings in Japan and the United States. *Cogn. Emot.* 14 93–124. 10.1080/026999300379003

[B35] Koc M. Gulyagci S. (2013). Facebook addiction among Turkish college students: the role of psychological health, demographic, and usage characteristics. *Cyberpsychol. Behav. Soc. Netw.* 16 279–284. 10.1089/cyber.2012.0249 23286695

[B36] Krasnova H. Wenninger H. Widjaja T. Buxmann P. (2013). Envy on Facebook: a hidden threat to users’ life satisfaction? *Wirtschaftsinformatik* 92 1–16.

[B37] Kraut R. Burke M. (2015). Internet use and psychological well-being: effects of Activity and Audience. *Commun. ACM* 58 94–100. 10.1145/2739043

[B38] Kross E. Verduyn P. Demiralp E. Park J. Lee D. S. Lin N. (2013). Facebook use predicts declines in subjective well-being in young adults. *PLoS One* 8:e69841 10.1371/journal.pone.0069841 PMC3743827 23967061

[B39] Liu D. Baumeister R. F. (2016). Social networking online and personality of self-worth: a meta-analysis. *J. Res. Pers.* 64 79–89. 10.1016/j.jrp.2016.06.024

[B40] Ljepava N. Orr R. R. Locke S. Ross C. (2013). Personality and social characteristics of Facebook non-users and frequent users. *Comput. Hum. Behav.* 29 1602–1607. 10.1016/j.chb.2013.01.026

[B41] Morf C. C. Rhodewalt F. (2001). Unraveling the paradoxes of narcissism: a dynamic self-regulatory processing model. *Psychol. Inq.* 12 177–196. 10.1207/S15327965PLI1204_1

[B42] Nadkarni A. Hofmann S. G. (2012). Why do people use Facebook? *Pers. Individ. Dif.* 52 243–249. 10.1016/j.paid.2011.11.007 22544987 PMC3335399

[B43] Ong E. Y. L. Ang R. P. Ho J. Lim J. C. Y. Goh D. H. Lee C. S. (2011). Narcissism, extraversion and adolescents’ self-presentation on Facebook. *Pers. Individ. Dif.* 50 180–185. 10.1016/j.paid.2010.09.022

[B44] Paulhus D. L. Robins R. W. Trzesniewski K. H. Tracy J. L. (2004). Two replicable suppressor situations in personality research. *Multivar. Behav. Res.* 39 303–328. 10.1207/s15327906mbr3902_7 26804578

[B45] Raskin R. Terry H. (1988). A principal-components analysis of the Narcissistic Personality Inventory and further evidence of its construct validity. *J. Pers. Soc. Psychol.* 54 890–902. 10.1037/0022-3514.54.5.890 3379585

[B46] Rhodewalt F. Madrian J. C. Cheney S. (1998). Narcissism, self-knowledge organization, and emotional reactivity: the effect of daily experiences on self-esteem and affect. *Pers. Soc. Psychol. Bull.* 24 75–87. 10.1177/0146167298241006

[B47] Rhodewalt F. Morf C. C. (1998). On self-aggrandizement and anger: a temporal analysis of narcissism and affective reactions to success and failure. *J. Pers. Soc. Psychol.* 74 672–685. 10.1037/0022-3514.74.3.672 9523411

[B48] Rose P. (2002). The happy and unhappy faces of narcissism. *Pers. Individ. Dif.* 33 379–391. 10.1016/S0191-8869(01)00162-3

[B49] Rosenberg M. (1965). *Society and the Adolescent Self-Image.* Princeton, NJ: Princeton University Press.

[B50] Rosenthal S. A. Hooley J. M. (2010). Narcissism assessment in social–personality research: does the association between narcissism and psychological health result from a confound with self-esteem? *J. Res. Pers.* 44 453–465. 10.1016/j.jrp.2010.05.008

[B51] Ross C. Orr E. S. Sisic M. Arseneault J. M. Simmering M. G. Orr R. R. (2009). Personality and motivations associated with Facebook use. *Comput. Hum. Behav.* 25 578–586. 10.1016/j.chb.2008.12.024

[B52] Ryan T. Xenos S. (2011). Who uses Facebook? An investigation into the relationship between the Big Five, shyness, narcissism, loneliness, and Facebook usage. *Comput. Hum. Behav.* 27 1658–1664. 10.1016/j.chb.2011.02.004

[B53] Sedikides C. Rudich E. A. Gregg A. P. Kumashiro M. Rusbult C. (2004). Are normal narcissists psychologically healthy? Self-esteem matters. *J. Pers. Soc. Psychol.* 87 400–416. 10.1037/0022-3514.87.3.400 15382988

[B54] Shi Y. Luo Y. L. L. Liu Y. Yang Z. (2018). Affective experience on social networking sites predicts psychological well-Being offline. *Psychol. Rep.* 10.1177/0033294118789039 [Epub ahead of print]. 30080110

[B55] Shi Y. Luo Y. L. L. Yang Z. Liu Y. Cai H. (2014). *The Development and Validation of the Social Network Sites (SNSs) Usage Questionnaire. Social Computing and Social Media.* Berlin: Springer, 113–124. 10.1007/978-3-319-07632-4_11

[B56] Taylor S. E. Brown J. D. (1988). Illusion and well-being: a social psychological perspective on mental health. *Psychol. Bull.* 103 193–210. 10.1037/0033-2909.103.2.193 3283814

[B57] Toma C. L. (2013). Feeling better but doing worse: effects of Facebook self-presentation on implicit self-esteem and cognitive task performance. *Media Psychol.* 16 199–220. 10.1080/15213269.2012.762189

[B58] Toma C. L. Hancock J. T. (2013). Self-affirmation underlies Facebook use. *Pers. Soc. Psychol. Bull.* 39 321–331. 10.1177/0146167212474694 23359086

[B59] Tritt S. M. Ryder A. G. Ring A. J. Pincus A. L. (2010). Pathological narcissism and the depressive temperament. *J. Affect. Disord.* 122 280–284. 10.1016/j.jad.2009.09.006 19800134

[B60] Uchida Y. Norasakkunkit V. Kitayama S. (2004). Cultural constructions of happiness: theory and empirical evidence. *J. Happiness Stud.* 5 223–239. 10.1007/s10902-004-8785-9

[B61] Van Schuur W. H. Kruijtbosch M. (1995). Measuring subjective well-being: unfolding the Bradburn affect balance scale. *Soc. Indicat. Res.* 36 49–74. 10.1007/BF01079396

[B62] Wang J. L. Jackson L. A. Zhang D. J. Su Z. Q. (2012). The relationships among the Big Five Personality factors, self-esteem, narcissism, and sensation-seeking to Chinese University students’ uses of social networking sites (SNSs). *Comput. Hum. Behav.* 28 2313–2319. 10.1016/j.chb.2012.07.001

[B63] Watson P. J. Biderman M. D. (1993). Narcissistic Personality Inventory factors, splitting, and self-consciousness. *J. Pers. Assess.* 61 41–57. 10.1207/s15327752jpa6101_4 8377102

[B64] Winter S. Neubaum G. Eimler S. C. Gordon V. Theil J. Herrmann J. (2014). Another brick in the Facebook wall–How personality traits relate to the content of status updates. *Comput. Hum. Behav.* 34 194–202. 10.1016/j.chb.2014.01.048

[B65] Wise K. Alhabash S. Park H. (2010). Emotional responses during social information seeking on Facebook. *Cyberpsychol. Behav. Soc. Netw.* 13 555–562. 10.1089/cyber.2009.0365 20950180

