# Supplementary material for: Do Narcissists Enjoy Visiting Social Networking Sites? It Depends on How Adaptive They Are
Source: Front Psychol. 2018 Sep 19;9:1739. doi: 10.3389/fpsyg.2018.01739 (PMC6156359; doi:10.3389/fpsyg.2018.01739)
Supplement: Supplementary file 1 [file Table_1.DOCX]

**Online Supplementary Material**

**Narcissism predicting SNS affective experience with age and gender controlled**

We included age and gender as additional predictors in the regression model (Study 1) and hierarchical linear model (Study 2). We found that neither of them was predicative of SNS affect (Table S1, S2). More important, the effects of adaptive and maladaptive narcissism remained almost the same when including age and gender in the model. Taken together, demographic factors such as gender and age were irrelevant to the present studies. As we have no a-priori reason to include demographic factors in the analyses, we presented results without controlling them in the main text. We included results with age and gender controlled here.

**Table S1. Unstandardized coefficients (B) of SNS affective experience regressed on narcissism and self-esteem with age and gender controlled (Study 1).**

| Predictor | *B* | *SE* | *p* |
| --- | --- | --- | --- |
| *Model 1* |  |  |  |
| Gender | .04 | .11 | .754 |
| Age | .03 | .02 | .117 |
| *Model 2* |  |  |  |
| Gender | .01 | .11 | .906 |
| Age | .02 | .02 | .232 |
| Adaptive narcissism | .06 | .02 | .012 |
| Maladaptive narcissism | -.06 | .02 | .010 |
| *Model 3* |  |  |  |
| Gender | .02 | .11 | .893 |
| Age | .02 | .02 | .228 |
| Adaptive narcissism | .06 | .02 | .012 |
| Maladaptive narcissism | -.06 | .02 | .011 |
| SNS use frequency | .01 | .05 | .803 |
| *Model 4* |  |  |  |
| Gender | .04 | .11 | .686 |
| Age | .01 | .02 | .480 |
| Adaptive narcissism | .02 | .03 | .381 |
| Maladaptive narcissism | -.04 | .02 | .049 |
| SNS use frequency | .01 | .05 | .897 |
| Self-esteem | .41 | .12 | .001 |

*Note*. SE = standard error.

**Table S2. Unstandardized coefficients (B) of SNS affective experience regressed on narcissism, self-esteem, and SNS feature usage with age and gender controlled (Study 2).**

| Predictor | *B* | *SE* | *p* |
| --- | --- | --- | --- |
| *Model 1* |  |  |  |
| Gender | -.01 | .08 | .890 |
| Age | .00 | .02 | .943 |
| *Model 2* |  |  |  |
| Gender | -.09 | .08 | .260 |
| Age | .01 | .02 | .778 |
| Adaptive narcissism | .07 | .02 | .000 |
| Maladaptive narcissism | -.03 | .01 | .015 |
| *Model 3* |  |  |  |
| Gender | -.08 | .08 | .286 |
| Age | .01 | .02 | .748 |
| Adaptive narcissism | .08 | .02 | .000 |
| Maladaptive narcissism | -.04 | .01 | .010 |
| General use in SNS | ..04 | .03 | .264 |
| Social interaction in SNS | .06 | .03 | .071 |
| Self-presentation in SNS | -.05 | .05 | .313 |
| *Model 4* |  |  |  |
| Gender | -.06 | .08 | .409 |
| Age | -.01 | .02 | .774 |
| Adaptive narcissism | .04 | .02 | .014 |
| Maladaptive narcissism | -.03 | .01 | .013 |
| General use in SNS | .03 | .03 | .334 |
| Social interaction in SNS | .06 | .03 | .070 |
| Self-presentation in SNS | -.04 | .05 | .388 |
| Self-esteem | .50 | .09 | .000 |

*Note*. SE = standard error.

**Additional analyses for Study 2**

In Study 2, we recruited twins. As twins are nested in pairs, they are not independent participants. We utilized the HLM in the main text to adjust the interdependence between twins. Here, we randomly select one sibling from each twin pair to ensure the data independence in a different way (Sample 1). We also replicated the analysis with the left twin sample (Sample 2). Participants who stated they did not visit SNS at all were excluded from the analysis, resulting in 208 individuals in Sample 1 (112 females) and 200 individuals in Sample 2 (113 female).

The results based on the two samples were very similar. Adaptive narcissism was positively associated with SNS affective experience on SNS, whereas maladaptive narcissism was negatively associated with SNS affective experience (Table S3). The only difference existed in the pattern for SNS activity, which is not surprising as previous literatures also presented an inconsistent relationship between well-being and SNS activity (Hu, Kim, Siwek & Wilder, 2017; Shi, Luo, Liu, & Yang, in press; Verduyn, Ybarra, Résibois, Jonides & Kross, 2017).

**Table S3. Standardized coefficients (beta) of SNS affective experience regressed on narcissism, self-esteem, and SNS feature usage.**

| **Predictor** | **Sample 1** | **Sample 2** |
| --- | --- | --- |
| *Model 1* |  |  |
| Adaptive narcissism | .20 ^**^ | .29 ^***^ |
| Maladaptive narcissism | -.14^+^ | -.18^*^ |
| *Model 2* |  |  |
| Adaptive narcissism | .21^**^ | .31^***^ |
| Maladaptive narcissism | -.19^*^ | -.16^+^ |
| General use in SNS | .16^*^ | -.05 |
| Social interaction in SNS | .02 | .24^**^ |
| Self-presentation in SNS | -.01 | -.10 |
| *Model 3* |  |  |
| Adaptive narcissism | .12 | .17^*^ |
| Maladaptive narcissism | -.17^*^ | -.14^+^ |
| General use in SNS | 12 | -.05 |
| Social interaction in SNS | .03 | .21^**^ |
| Self-presentation in SNS | -.03 | -.06 |
| Self-esteem | .27^***^ | .31^***^ |

*Note*. ^+^*p* < .1; ^*^*p* < .05; ^**^*p* < .01; ^***^*p* < .001.

**References**

Hu, X., Kim, A., Siwek, N. & Wilder, D. (2017). The Facebook paradox: Effects of Facebooking on individuals’ social relationships and psychological well-being. *Frontiers in Psychology. 8*:87. doi:10.3389/fpsyg.2017.00087

Shi, Y., Luo, Y. L.L., Liu, Y.,& Yang, Z. (in press). Affective Experience on Social Networking Sites Predicts Psychological Well-Being Offline. *Psychological Report*.

Verduyn, P., Ybarra, O., Résibois, M., Jonides, J., & Kross, E. (2017). Do social network sites enhance or undermine subjective well-being? A critical review. *Social Issues and Policy Review, 11*, 274-302. doi: 10.1111/sipr.12033
